# Supplementary material for: Synthetic reconstruction of the hunchback promoter specifies the role of Bicoid, Zelda and Hunchback in the dynamics of its transcription
Source: eLife. 2022 Apr 1;11:e74509. doi: 10.7554/eLife.74509 (PMC8975551; doi:10.7554/eLife.74509)
Supplement: Supplementary file 1. — The sequences are shown in modules, as arranged in Figure 1 in the main text. The TATA boxes of hb-P2 and HSBG promoter are highlighted in bold. In the binding array sequences, the binding sites for each protein are highlighted in grey (Bcd), green (Hb) and yellow (Zelda). For clarity, the binding array sequences are shown up to the TATA box of HSBG promoter. [file elife-74509-supp1.docx]

# Supplementary File 1

| Name | Sequence |
| --- | --- |
| hb-P2  (*hb* intron included) | ctgtcgactcctgaccaacgtaatccccatagaaaaccggtggaaaattcgcagctcgctgctaagctggccatccgctaagctcccggatcatccaaatccaagtgcgcataattttttgtttctgctctaatccagaatggatcaagagcgcaatcctcaatccgcgatccgtgatcctcgattcccgaccgatccgcgacctgtacctgacttcccgtcacctctgcccatctaatcccttgacgcgtgcatccgtctacctgagcga**tatataaa**ctaatgcctgttgcaattgttcagtcagtcacgagtttgttaccactgcgacaacacaacagaagcagcaccaataatatacttgcaaatccttacgaaaatcccgacaaatttggaatatacttcgatacaatcgcaatcatacgcactgagcggccacgaaacggtaggatattgttagccattaccaagtgtctccattttgaacacaaaatcactcaaatcgccttcagggggtgggtgccgcccagccacccctgacgtattttttgttaggggtggtgccgcaagcacaccaaaaaaagagaaaaaaaaaataaaagcgaggaaaaataaaatgaaaaacaagcggaaaaaaagaggaaaaaactcgacgcaggcgcagtgcatgaatgaataaatgaatatgcccactaaccccactctctctgttttcttatccattacagccgtctagagccgccaagg |
| iRFP | atggcgcgtaaggtcgatctcacctcctgcgatcgcgagccgatccacatccccggcagcattcagccgtgcggCTGTCTCctagcctgcgacgcgcaggcggtgcggatcacgcgcattacggaaaatgccggcgcgttctttggacgcgaaactccgcgggtcggtgagctactcgccgattacttcggcgagaccgaagcccatgcgctgcgcaacgcactggcgcagtcctccgatccaaagcgaccggcgctgatcttcggttggcgcgacggcctgaccggccgcaccttcgacatctcactgcatcgccatgacggtacatcgatcatcgagttcgagcctgcggcggccgaacaggccgacaatccgctgcggctgacgcggcagatcatcgcgcgcaccaaagaactgaagtcgctcgaagagatggccgcacgggtgccgcgctatctgcaggcgatgctcggctatcaccgcgtgatgttgtaccgcttcgcggacgacggctccgggatggtgatcggcgaggcgaagcgcagcgacctcgagagctttctcggtcagcactttccggcgtcgctggtcccgcagcaggcgcggctactgtacttgaagaacgcgatccgcgtggtctcggattcgcgcggcatcagcagccggatcgtgcccgagcacgacgcctccggcgccgcgctcgatctgtcgttcgcgcacctgcgcagcatctcgccctgccatctcgaatttctgcggaacatgggcgtcagcgcctcgatgtcgctgtcgatcatcattgacggcacgctatggggattgatcatctgtcatcattacgagccgcgtgccgtgccgatggcgcagcgcgtcgcggccgaaatgttcgccgacttcttatcgctgcacttcaccgccgcccaccaccaacgctaa |
| MS2 | GGATCCTACGGTACTTATTGCCAAGAAAGCACGAGCATCAGCCGTGCCTCAATGTCGAATCTGCAAACGACGACGATCACGCGTCGCTCCAGTATTCCAGGGTTCATCAGATCCTACGGTACTTATTGCCAAGAAAGCACGAGCATCAGCCGTGCCTCAATGTCGAATCTGCAAACGACGACGATCACGCGTCGCTCCAGTATTCCAGGGTTCATCAGATCCTACGGTACTTATTGCCAAGAAAGCACGAGCATCAGCCGTGCCTCAATGTCGAATCTGCAAACGACGACGATCACGCGTCGCTCCAGTATTCCAGGGTTCATCAGATCCTACGGTACTTATTGCCAAGAAAGCACGAGCATCAGCCGTGCCTCAATGTCGAATCTGCAAACGACGACGATCACGCGTCGCTCCAGTATTCCAGGGTTCATCAGATCCTACGGTACTTATTGCCAAGAAAGCACGAGCATCAGCCGTGCCTCAATGTCGAATCTGCAAACGACGACGATCACGCGTCGCTCCAGTATTCCAGGGTTCATCAGATCCTACGGTACTTATTGCCAAGAAAGCACGAGCATCAGCCGTGCCTCAATGTCGAATCTGCAAACGACGACGATCACGCGTCGCTCCAGTATTCCAGGGTTCATCAGATCCTACGGTACTTATTGCCAAGAAAGCACGAGCATCAGCCGTGCCTCAATGTCGAATCTGCAAACGACGACGATCACGCGTCGCTCCAGTATTCCAGGGTTCATCAGATCCTACGGTACTTATTGCCAAGAAAGCACGAGCATCAGCCGTGCCTCAATGTCGAATCTGCAAACGACGACGATCACGCGTCGCTCCAGTATTCCAGGGTTCATCAGATCCTACGGTACTTATTGCCAAGAAAGCACGAGCATCAGCCGTGCCTCAATGTCGAATCTGCAAACGACGACGATCACGCGTCGCTCCAGTATTCCAGGGTTCATCAGATCCTACGGTACTTATTGCCAAGAAAGCACGAGCATCAGCCGTGCCTCAATGTCGAATCTGCAAACGACGACGATCACGCGTCGCTCCAGTATTCCAGGGTTCATCAGATCCTACGGTACTTATTGCCAAGAAAGCACGAGCATCAGCCGTGCCTCAATGTCGAATCTGCAAACGACGACGATCACGCGTCGCTCCAGTATTCCAGGGTTCATCAGATCCTACGGTACTTATTGCCAAGAAAGCACGAGCATCAGCCGTGCCTCAATGTCGAATCTGCAAACGACGACGATCACGCGTCGCTCCAGTATTCCAGGGTTCATCAGATCC |
| HSBG promoter | ACCGCCGGAG**TATAAATA**GAGGCGCTTCGTCTACGGAGCGACAATTCAATTCAAACAAG**C**AAAGTGAACACGTCGCTAAGCGAAAGCTAAGCAAATAAACAAGCGCAGCTGAACAAGCTaaacaatcggggtacggctagca |
| B6 | TCGACTCATGGGATTAGACTCGAGGGATTAGACCGGGATTAGAACCTGGGGATCGGGGATTAGACTCGAGGGATTAGACCGGGATTAGAGGATCCAAGCTTATCGATTTCGAACCCTCGACCGCCGGAG**TATAAATA** |
| B9 | TCGACTCATGGGATTAGACTCGAGGGATTAGACCGGGATTAGAACCTGGGGATCGGGGATTAGACTCGAGGGATTAGACCGGGATTAGAGGATCCATGGGATTAGACTCGAGGGATTAGACCGGGATTAGAGGGATCCAAGCTTATCGATTTCGAACCCTCGACCGCCGGAG**TATAAATA** |
| B12 | TCGACTCATGGGATTAGACTCGAGGGATTAGACCGGGATTAGAACCTGGGGATCGGGGATTAGACTCGAGGGATTAGACCGGGATTAGAGGATCCATGGGATTAGACTCGAGGGATTAGACCGGGATTAGAACCTGGGGATCGGGGATTAGACTCGAGGGATTAGACCGGGATTAGAGGGATCCAAGCTTATCGATTTCGAACCCTCGACCGCCGGAG**TATAAATA** |
| H6 | TCGGTACCATAGTTTTTTGAGTATCGATAGTTTTTTGAGTCCATATTTTTTGAGTACTCATAGTTTTTTGAGTATCGATAGTTTTTTGAGTCCATATTTTTTGAGTGTCGACGGTATCGATAAGCGGATCCAAGCTTATCGATTTCGAACCCTCGACCGCCGGAG**TATAAATA** |
| H6B6 | TCGGTACCACTCAAAAAATATGGACTCAAAAAACTATCGATACTCAAAAAACTATGAGTACTCAAAAAATATGGACTCAAAAAACTATCGATACTCAAAAAACTATGGTACCGTACCCCGTCGACTCATGGGATTAGACTCGAGGGATTAGACCGGGATTAGAACCTGGGGATCGGGGATTAGACTCGAGGGATTAGACCGGGATTAGAGGATCCAAGCTTATCGATTTCGAACCCTCGACCGCCGGAG**TATAAATA** |
| Z2 | TCATGGATCTCAGGTAGTAACTGACCAGGTAGGAGGATCCAAGCTTATCGATTTCGAACCCTCGACCGCCGGAG**TATAAATA** |
| Z2B6 | TCATGGATCCAGGTAGTAACTGACCAGGTAGCTATGGTACCGTACCCCGTCGACTCATGGGATTAGACTCGAGGGATTAGACCGGGATTAGAACCTGGGGATCGGGGATTAGACTCGAGGGATTAGACCGGGATTAGAGGATCCAAGCTTATCGATTTCGAACCCTCGACCGCCGGAG**TATAAATA** |
| Z6 | TCATGGATCCAGGTAGATATCGCACAGGTAGCGATCATACAGGTAGCCTAGATCCAGGTAGTCAATGATCAGGTAGTAACTGACCAGGTAGGATCCAAGCTTATCGATTTCGAACCCTCGACCGCCGGAG**TATAAATA** |
| Oligo for 5’ cut Fw | 5’-GTCGGTCTAATTGATTCCTAAATT-3’ |
| Oligo for 5’ cut Rv | 5’-AAACAATTTAGGAATCAATTAGAC-3’ |
| Oligo for 3’ cut Fw | 5’-GTCGGAATGAACGAAAACAGTATC-3’ |
| Oligo for 3’ cut Rv | 5’-AAACGATACTGTTTTCGTTCATTC-3’ |
| Bcdnull_5HR_fw | 5’- TAAGAGACGTATAGGAGACCTATAGTGTCTTCGGGGCCGATTCGAAAACT  TTCTGCTGCC-3’ |
| Bcdnull_5HR_rv | 5’-TTTAACGTACGTCACAATATGATTATCTTTCTAGGGTTAATCAATTAGACAAGTG  TCGAATGTTTAATTTG-3’ |
| Bcdnull_DsRed_fw | 5’- ACAAAAATTCAAATTAAACATTCGACACTTGTCTAATTGATTAACCCTAGAAA  GATAATC-3’ |
| Bcdnull_DsRed_rv | 5’- ATTTTTAAATTCATAAGATTTTCGGGAAAACCAGATACTGTTAACCCTAG  AAAGATAGTC-3’ |
| Bcdnull_3HR_fw | 5’-gcgcGCTCTTCgTAACAGTATCTGGTTTTCCCGAA-3’ |
| Bcdnull_3HR_rv | 5’-tataGCTCTTCaCGGCTGGTGAAGGCAGTCCGTGA-3’ |

**Supplementary File 1. Promoter sequences of hb-P2, synthetic MS2 reporters and oligonucleotides required for generating the *Δ bcd* molecular null allele.** The sequences are shown in modules, as arranged in Figure 1 in the main text. The TATA boxes of hb-P2 and HSBG promoter are highlighted in bold. In the binding array sequences, the binding sites for each protein are highlighted in grey (Bcd), green (Hb) and yellow (Zelda). For clarity, the binding array sequences are shown up to the TATA box of HSBG promoter.
