# Supplementary material for: Synthetic reconstruction of the hunchback promoter specifies the role of Bicoid, Zelda and Hunchback in the dynamics of its transcription
Source: eLife. 2022 Apr 1;11:e74509. doi: 10.7554/eLife.74509 (PMC8975551; doi:10.7554/eLife.74509)
Supplement: Supplementary file 2. — For the fraction of expression nuclei, also shown is the time to reach the final activation decision boundary ( ± 2 %EL) starting from the detection the first spot (~225 s) after mitosis. [file elife-74509-supp2.docx]

# Supplementary File 2

|  | Fraction of expressing nuclei | | | Fraction of active loci  at steady state $P_{Spot}$ | | |
| --- | --- | --- | --- | --- | --- | --- |
|  | Boundary position (%EL) | Boundary width (%EL) | Converging time (s) | Boundary position (%EL) | Boundary width (%EL) | Pattern  Sharpness  (%EL)^-1^ |
| hb-P2 | 45.0 ± 0.3 | 11.0 ± 1.6 | 225 ± 25 | 40.1 ± 0.4 | 16.8 ± 2.0 | 0.33 ± 0.04 |
| B6 | 37.1 ± 1.0 | 25.6 ± 3.6 | 425± 25 | 34.4 ± 1.0 | 24.7 ± 5.6 | 0.22 ± 0.05 |
| B9 | 44.9 ± 0.4 | 17.6 ± 2.2 | 475± 25 | 42.7 ± 0.5 | 18.9 ± 2.0 | 0.30 ± 0.07 |
| B12 | 45.8 ± 0.5 | 16.4 ± 2.5 | 325 ± 25 | 44.0 ± 0.7 | 19.6 ± 6.9 | 0.29 ± 0.07 |
| H6B6 | 40.2 ± 0.4 | 13.8 ± 1.8 | 325 ± 25 | 36.7 ± 0.4 | 18.9 ± 2.0 | 0.30 ± 0.03 |
| Z2B6 | 54.6 ± 0.7 | 19.4 ± 3.2 | 300 ± 25 | 49.4 ± 1.4 | 19.6 ± 6.9 | 0.29 ± 0.12 |

**Supplementary File 2.** Position and width of the gene expression boundary based on fraction of expression nuclei feature (Figure 1H and Figure 3G) and fraction of active loci $P_{spot}$ feature (Figure 2A and Figure 3H) in the steady window (600-800s into nc13) for hb-P2 and synthetic reporters in Bcd-2X, shown with 95% confidence interval. For the fraction of expression nuclei, also shown is the time to reach the final activation decision boundary (±2 %EL) starting from the detection the first spot (~225 s) after mitosis.
