# Supplementary material for: Synthetic reconstruction of the hunchback promoter specifies the role of Bicoid, Zelda and Hunchback in the dynamics of its transcription
Source: eLife. 2022 Apr 1;11:e74509. doi: 10.7554/eLife.74509 (PMC8975551; doi:10.7554/eLife.74509)
Supplement: Supplementary file 3. [file elife-74509-supp3.docx]

# Supplementary File 3

| **λ in µm** | **λ as % EL** | **Method of detection** | **Reference** |
| --- | --- | --- | --- |
| 125 | ~ 25 | live detection of the Bcd-eGFP fusion | (Abu-Arish et al., 2010) |
| 100 | ~ 20 | fixed embryos, antibody against the wt Bicoid protein | (Houchmandzadeh et al., 2002) |
| 100 | ~20 | Live detection of Bcd-eGFP | (Gregor et al., 2007b) |
| 100 | 20 | Fixed embryos, antibodies against the wt Bicoid protein at nc13 | (Xu et al., 2015) |
|  | 19,3 | live detection of the Bcd-eGFP fusion | (Liu et al., 2013) (Fig. S4) |
|  | 18,2 | live detection of the Bcd-Venus fusion | (Liu et al., 2013) (Fig. S4) |
|  | 16.4 | fixed embryos, Bcd antibody against Bcd-eGFP | (Liu et al., 2013) (Fig. S4) |
| 89 | ~ 17.8 | Live detection of tandem-fluorescent protein timer fused to Bcd | (Durrieu et al., 2018) (p7) |

# Supplementary File 3. Estimated values of gradient decay length for the Bcd protein and Bcd fluorescently tagged protein gradients from previous studies.

**References**

Abu-Arish A, Porcher A, Czerwonka A, Dostatni N, Fradin C. 2010. High mobility of Bicoid captured by fluorescence correlation spectroscopy: Implication for the rapid establishment of its gradient. *Biophys J* **99**:33–35. doi:10.1016/j.bpj.2010.05.031

Durrieu L, Kirrmaier D, Schneidt T, Kats I, Raghavan S, Hufnagel L, Saunders TE, Knop M. 2018. Bicoid gradient formation mechanism and dynamics revealed by protein lifetime analysis. *Mol Syst Biol* **14**:e8355. doi:10.15252/msb.20188355

Gregor T, Wieschaus E, McGregor AP, Bialek W, Tank DW. 2007b. Stability and nuclear dynamics of the Bicoid morphogen gradient. *Cell* **130**:141–152. doi:10.1016/j.cell.2007.05.026

Houchmandzadeh B, Wieschaus E, Leibler S. 2002. Establishment of developmental precision and proportions in the early Drosophila embryo. *Nature* **415**:798–802. doi:10.1038/415798a

Liu F, Morrison AH, Gregor T. 2013. Dynamic interpretation of maternal inputs by the Drosophila segmentation gene network. *Proc Natl Acad Sci* **110**:6724–6729. doi:10.1073/pnas.1220912110

Xu H, Sepúlveda LA, Figard L, Sokac AM, Golding I. 2015. Combining protein and mRNA quantification to decipher transcriptional regulation. *Nat Methods* **12**:739–42. doi:10.1038/nmeth.3446
