# Supplementary material for: Synthetic reconstruction of the hunchback promoter specifies the role of Bicoid, Zelda and Hunchback in the dynamics of its transcription
Source: eLife. 2022 Apr 1;11:e74509. doi: 10.7554/eLife.74509 (PMC8975551; doi:10.7554/eLife.74509)
Supplement: Supplementary file 4. [file elife-74509-supp4.docx]

# Supplementary File 4

| Parameters | Value | Measurement method | Reference |
| --- | --- | --- | --- |
| $c_{A}$  (molecules/μm^3^) | ~33 | Imaging with Bcd-eGFP | (Gregor et al., 2007b) |
|  | ~84 | Extract using Bcd-eGFP imaging and FCS | (Abu-Arish et al., 2010) |
|  | ~210 | Comparison between immunostaining and imaging of Bcd-eGFP reveals ~ 2.5 time underestimation of Bcd concentration | (Liu et al., 2013) |
| $D$  (μm^2^/s) | <1 | Imaging with Bcd-eGFP using FRAP | (Gregor et al., 2007a) |
|  | ~7.4 | Diffusion coefficient of fast-diffusing Bcd population, extracted using FCS and Bcd-eGFP | (Abu-Arish et al., 2010) |
|  | ~4.6 | Average diffusion coefficient, extract using FCS and Bcd-eGFP | (Abu-Arish et al., 2010) |
| $a$  (nm) | 3 | Size for a single binding site of ~10bp |  |
|  | 0.3 | Size of a single nucleotide for exact match |  |

**Supplementary File 4.** Estimated values of Bcd concentration at the anterior ($c_{A}$), diffusion coefficient ($D$) and the size of the target for binding ($a$) from previous work.

# References

Abu-Arish A, Porcher A, Czerwonka A, Dostatni N, Fradin C. 2010. High mobility of Bicoid captured by fluorescence correlation spectroscopy: Implication for the rapid establishment of its gradient. *Biophys J* **99**:33–35. doi:10.1016/j.bpj.2010.05.031

Gregor T, Tank DW, Wieschaus E, Bialek W. 2007a. Probing the limits to positional information. *Cell* **130**:153–164. doi:10.1016/j.cell.2007.05.025

Gregor T, Wieschaus E, McGregor AP, Bialek W, Tank DW. 2007b. Stability and nuclear dynamics of the Bicoid morphogen gradient. *Cell* **130**:141–152. doi:10.1016/j.cell.2007.05.026

Liu F, Morrison AH, Gregor T. 2013. Dynamic interpretation of maternal inputs by the Drosophila segmentation gene network. *Proc Natl Acad Sci* **110**:6724–6729. doi:10.1073/pnas.1220912110
