# Supplementary material for: Synthetic reconstruction of the hunchback promoter specifies the role of Bicoid, Zelda and Hunchback in the dynamics of its transcription
Source: eLife. 2022 Apr 1;11:e74509. doi: 10.7554/eLife.74509 (PMC8975551; doi:10.7554/eLife.74509)
Supplement: Supplementary file 5. [file elife-74509-supp5.docx]

# Supplementary File 5

| $\lambda$ (%EL) | $c_{A}$ (1/μm^3^) | $D$ (μm^2^/s) | $a$ (nm) | $t_{bind}$ (s) | $T$ (min) |
| --- | --- | --- | --- | --- | --- |
| 15 | 33 | 7.4 | 3 | 26.9 | 7.5 |
|  |  |  | 0.3 | 268.8 | 74.7 |
|  |  | 4.6 | 3 | 43.2 | 12.0 |
|  |  |  | 0.3 | 432.4 | 120.1 |
|  | 84 | 7.4 | 3 | 10.6 | 2.9 |
|  |  |  | 0.3 | 105.6 | 29.3 |
|  |  | 4.6 | 3 | 17.0 | 4.7 |
|  |  |  | 0.3 | 169.9 | 47.2 |
|  | 210 | 7.4 | 3 | 4.2 | 1.2 |
|  |  |  | 0.3 | 42.2 | 11.7 |
|  |  | 4.6 | 3 | 6.8 | 1.9 |
|  |  |  | 0.3 | 67.9 | 18.9 |
| 20 | 33 | 7.4 | 3 | 13.0 | 3.6 |
|  |  |  | 0.3 | 129.5 | 36.0 |
|  |  | 4.6 | 3 | 20.8 | 5.8 |
|  |  |  | 0.3 | 208.3 | 57.9 |
|  | 84 | 7.4 | 3 | 5.1 | 1.4 |
|  |  |  | 0.3 | 50.9 | 14.1 |
|  |  | 4.6 | 3 | 8.2 | 2.3 |
|  |  |  | 0.3 | 81.8 | 22.7 |
|  | 210 | 7.4 | 3 | 2.0 | 0.6 |
|  |  |  | 0.3 | 20.4 | 5.7 |
|  |  | 4.6 | 3 | 3.3 | 0.9 |
|  |  |  | 0.3 | 32.7 | 9.1 |

**Supplementary File 5.** Bcd search time for the target site ($t_{bind}$) and the readout time ($T$) required for 10% error in Bcd concentration readout at hb-P2 boundary position (-4.9 %EL), calculated for different values of Bcd concentration at the anterior ($c_{A}$), diffusion coefficient ($D$) and targets size ($a$) in the Berg & Purcell limit (Berg and Purcell, 1977).

# References

Berg HC, Purcell EM. 1977. Physics of chemoreception. *Biophys J* **20**:193–219. doi:10.1016/S0006-3495(77)85544-6
