# Supplementary material for: Synthetic reconstruction of the hunchback promoter specifies the role of Bicoid, Zelda and Hunchback in the dynamics of its transcription
Source: eLife. 2022 Apr 1;11:e74509. doi: 10.7554/eLife.74509 (PMC8975551; doi:10.7554/eLife.74509)
Supplement: Supplementary file 6. — The fold changes are shown for different schemes of activation and values of K (Figure 2—figure supplement 3). The fold changes above the value calculated from the data (~4.5) are made bold. [file elife-74509-supp6.docx]

# Supplementary File 6

| Scheme | $K$ | $\frac{k_{ON}\left( B9 \right)}{k_{ON}(B6)}$ | $\frac{k_{ON}\left( B12 \right)}{k_{ON}(B6)}$ |
| --- | --- | --- | --- |
| Independent activation  $k_{ON}\left( S_{i} \right)=ik_{ON}(S_{1})$ | $1$ | 1.5 | 2 |
| Formation of transient *K*-mer  $k_{ON}\left( S_{i} \right)=\frac{i!}{K!\left( i-K \right)!}k_{ON}(S_{K})$ | $1$ | 1.5 | 2 |
|  | $2$ | 2.4 | 4.4 |
|  | $3$ | 4.2 | **11** |
|  | $4$ | **8.4** | **33** |
|  | $5$ | **21** | **132** |
|  | $6$ | **84** | **924** |
| Formation of stable K-mer  $k_{ON}\left( S_{i} \right)=k_{ON}(S_{K})$ | $0\leq K\leq6$ | 1 | 1 |

**Supplementary File 6.** Expected fold change in the activation rates in the anterior region (saturating Bcd concentration) $k_{ON}(S_{i=N})$ between B9 and B6 and between B12 and B6. The fold changes are shown for different schemes of activation and values of *K* (Figure 2-figure supplement 3). The fold changes above the value calculated from the data (~4.5) are made bold.
