# Supplementary material for: Synthetic reconstruction of the hunchback promoter specifies the role of Bicoid, Zelda and Hunchback in the dynamics of its transcription
Source: eLife. 2022 Apr 1;11:e74509. doi: 10.7554/eLife.74509 (PMC8975551; doi:10.7554/eLife.74509)
Supplement: Transparent reporting form [file elife-74509-transrepform1.pdf]

eLIFE
1st Floor
24 Hills Road
Cambridge CB2 1JP, UK
P 01223 855340
W elifesciences.org
T @elife

eLife's transparent reporting form

We encourage authors to provide detailed information *within their submission* to facilitate the interpretation and replication of experiments. Authors can upload supporting documentation to indicate the use of appropriate reporting guidelines for health-related research (see EQUATOR Network), life science research (see the BioSharing Information Resource), or the ARRIVE guidelines for reporting work involving animal research. Where applicable, authors should refer to any relevant reporting standards documents in this form.

If you have any questions, please consult our Journal Policies and/or contact us: editorial@elifesciences.org.

Sample-size estimation

- You should state whether an appropriate sample size was computed when the study was being designed
- You should state the statistical method of sample size computation and any required assumptions
- If no explicit power analysis was used, you should describe how you decided what sample (replicate) size (number) to use

Please outline where this information can be found within the submission (e.g., sections or figure legends), or explain why this information doesn't apply to your submission:

In this study, we analyzed movies of developing embryos with specific maternal and zygotic genotypes. The sample size is the number of nuclei captured within the imaging region of 300 µm (Antero-Posterior axis) by 100µ (Ventral Dorsal axis). This region in our cycle of interest (nc13) contains ~1000 nuclei. There is no explicit power analysis for this size. We consider this sample size is sufficient given the reproducibility of expression patterns found in this study (Fig. 1H, Fig. 2A,B and Fig. 3G, I) and from previous works (Lucas et al, 2018, Lucas et al, 2013, Garcia et al, 2013).

Replicates

- You should report how often each experiment was performed
- You should include a definition of biological versus technical replication
- The data obtained should be provided and sufficient information should be provided to indicate the number of independent biological and/or technical replicates
- If you encountered any outliers, you should describe how these were handled
- Criteria for exclusion/inclusion of data should be clearly stated
- High-throughput sequence data should be uploaded before submission, with a private link for reviewers provided (these are available from both GEO and ArrayExpress)

Please outline where this information can be found within the submission (e.g., sections or figure legends), or explain why this information doesn't apply to your submission:

eLife Sciences Publications, Ltd is a limited liability non-profit non-stock corporation incorporated in the State of Delaware, USA, with company number 5030732, and is registered in the UK with company number FC030576 and branch number BR015634 at the address 1st Floor, 24 Hills Road, Cambridge CB2 1JP | August 2014

In our study, the number of biological replicates refers to the number of embryos of a given genotype analyzed. The information is indicated in the figure legends. All embryos were taken except for the very rare ones experiencing aberrant development.
We analyzed 3 to 7 embryos for each genotype. Given the very high reproducibility of the expression patterns between samples of the same genotype (Fig. 1H, Fig. 2A,B and Fig. 3G, I), we consider this size is sufficient to capture the features of expression patterns within the acceptable error of 2 % embryo length (a nucleus length).
As our data is obtained from real-time experiments, we don't use technical replicates in our study.

Statistical reporting

- Statistical analysis methods should be described and justified
- Raw data should be presented in figures whenever informative to do so (typically when N per group is less than 10)
- For each experiment, you should identify the statistical tests used, exact values of N, definitions of center, methods of multiple test correction, and dispersion and precision measures (e.g., mean, median, SD, SEM, confidence intervals; and, for the major substantive results, a measure of effect size (e.g., Pearson's r, Cohen's d)
- Report exact p-values wherever possible alongside the summary statistics and 95% confidence intervals. These should be reported for all key questions and not only when the p-value is less than 0.05.

Please outline where this information can be found within the submission (e.g., sections or figure legends), or explain why this information doesn't apply to your submission:

In Fig. 1, 2 and 3, pattern features are shown with 95% confidence intervals (indicated in Figure legends).
In Fig. 5, when distinguishing the models' goodness of fit to the data, we use a likelihood ratio test with a threshold for p-value equal 0.05.

(For large datasets, or papers with a very large number of statistical tests, you may upload a single table file with tests, Ns, etc., with reference to sections in the manuscript.)

Group allocation

- Indicate how samples were allocated into experimental groups (in the case of clinical studies, please specify allocation to treatment method); if randomization was used, please also state if restricted randomization was applied
- Indicate if masking was used during group allocation, data collection and/or data analysis

Please outline where this information can be found within the submission (e.g., sections or figure legends), or explain why this information doesn't apply to your submission:

This information doesn't apply to our submission because we did not allocate samples in experimental groups.

Additional data files ("source data")

- We encourage you to upload relevant additional data files, such as numerical data that are represented as a graph in a figure, or as a summary table

- Where provided, these should be in the most useful format, and they can be uploaded as "Source data" files linked to a main figure or table
- Include model definition files including the full list of parameters used
- Include code used for data analysis (e.g., R, MatLab)
- Avoid stating that data files are "available upon request"

Please indicate the figures or tables for which source data files have been provided:

All the movies used are deposited at Zenodo and are accessible through a community link: https://zenodo.org/communities/hb-synthetic/

Each dataset (several movies of embryos with the same genotype) are referenceable and can be accessed through their individual DOI:
hb-P2: https://doi.org/10.5281/zenodo.5361599
B6: https://doi.org/10.5281/zenodo.5457893
B9: https://doi.org/10.5281/zenodo.5457944
B12: https://doi.org/10.5281/zenodo.5458309
H6: https://doi.org/10.5281/zenodo.5459332
H6B6: https://doi.org/10.5281/zenodo.5458777
Z6: https://doi.org/10.5281/zenodo.5459338
Z2: https://doi.org/10.5281/zenodo.5459336
Z2B6: https://doi.org/10.5281/zenodo.5459314
bcd1X(delta)-hb-P2: https://doi.org/10.5281/zenodo.5463611
bcd1X(delta)-B9: https://doi.org/10.5281/zenodo.5463618
bcd1X(bcdE1)-hb-P2: https://doi.org/10.5281/zenodo.5464256
bcd1X(bcdE1)-B6: https://doi.org/10.5281/zenodo.5464655
bcd1X(bcdE1)-B9: https://doi.org/10.5281/zenodo.5465647
bcd1X(bcdE1)-B12: https://doi.org/10.5281/zenodo.5466741
bcd1X(bcdE1)-H6B6: https://doi.org/10.5281/zenodo.5466785
bcd1X(bcdE1)-Z2B6: https://doi.org/10.5281/zenodo.5466823
B6-hbpromoter: https://doi.org/10.5281/zenodo.5473374
hb-P2-II: https://doi.org/10.5281/zenodo.5477862
hb-P2-III: https://doi.org/10.5281/zenodo.5477926

eLife Sciences Publications, Ltd is a limited liability non-profit non-stock corporation incorporated in the State of Delaware, USA, with company number 5030732, and is registered in the UK with company number FC030576 and branch number BR015634 at the address 1st Floor, 24 Hills Road, Cambridge CB2 1JP | August 2014
